# Validation and reliability of the Dutch version of the EORTC QLQ-BLM30 module for assessing the health-related quality of life of patients with muscle invasive bladder cancer

**DOI:** 10.1186/s12955-022-02064-z

**Published:** 2022-12-29

**Authors:** T. M. Ripping, E. Rammant, J. A. Witjes, N. K. Aaronson, M. van Hemelrijck, L. M. C. van Hoogstraten, BlaZIB study group, J. Boormans, C. A. Goossens, A. G. van der Heijden, M. C. C. M. Hulshof, G. J. L. H. van Leenders, A. M. van Leliveld, R. P. Meijer, R. J. A. van Moorselaar, S. F. Mulder, R. I. Nooter, J. L. Noteboom, J. R. Oddens, T. M. de Reijke, B. W. G. van Rhijn, J. G. H. van Roermund, T. J. Smilde, G. W. J. Vanderbosch, B. P. Wijsman, L. A. Kiemeney, K. K. H. Aben

**Affiliations:** 1 Department of Research and Development, Netherlands Comprehensive Cancer Organisation, PO Box 19079, 3501 DB Utrecht, the Netherlands; 2 King’s College London, Faculty of Life Sciences & Medicine, Translational Oncology & Urology Research (TOUR), London, UK; 3 Department of Human Structure and Repair, Ghent University, Ghent, Belgium; 4 Department of Urology, Radboud University Medical Center, Nijmegen, the Netherlands; 5 Division of Psychosocial Research & Epidemiology, The Netherlands Cancer Institute, Amsterdam, The Netherlands; 6 Department for Health Evidence, Radboud University Medical Center, Nijmegen, the Netherlands

**Keywords:** Bladder cancer, Quality of life, Validation studies, EORTC questionnaire, Patient-reported outcomes measures

## Abstract

**Background:**

Quality of Life (QoL) of bladder cancer patients has been largely neglected. This is partly due to the lack of well-validated QoL questionnaires. The aim of this study is to examine the structural validity, reliability (i.e., internal consistency and test-retest reliability), construct validity (i.e., divergent validity and known group validity) and responsiveness of the Dutch version of the European Organisation for Research and Treatment of Cancer QoL questionnaire for muscle invasive bladder cancer (EORTC-QLQ-BLM30).

**Methods:**

Patients with newly diagnosed muscle invasive bladder cancer (MIBC) participating in the population-based ‘Blaaskankerzorg In Beeld’ (BlaZIB) study who completed the EORTC-QLQ-BLM30 at baseline were included. BlaZIB is a Dutch nationwide population-based prospective cohort study collecting clinical data and QoL data of bladder cancer patients. QoL is assessed with a self-administered questionnaire at four points in time: 6 weeks (baseline), 6 months, 12 months and 24 months after diagnosis. Confirmatory factor analysis and multitrait scaling analysis were used to investigate and adapt the scale structure. Reliability, construct validity and responsiveness of the revised scales were evaluated.

**Results:**

Of the 1542 patients invited to participate, 650 patients (42.2%) completed the QLQ-BLM30 at baseline. The questionnaire’s scale structure was revised into seven scales and eight single items. Internal consistency and test-reliability were adequate for most scales (Cronbach’s α ≥0.70 and intraclass correlation coefficient ≥ 0.70, respectively), with the exception of the revised urostomy problem scale and abdominal bloating and flatulence scale. The questionnaire exhibited little overlap with the EORTC-QLQ-C30: all correlations were < 0.40, except for the correlation between emotional function (QLQ-C30) and future worries (QLQ-BLM30). The questionnaire was able to distinguish between patient subgroups formed on the basis of physical function, but not – as hypothesized– based on stage. Changes in health due to treatment were captured by the questionnaire, indicating that the questionnaire is responsive to change.

**Conclusions:**

This study shows that the adapted scale structure of the EORTC-QLQ-BLM30 generally exhibits good measurement properties in Dutch patients, but needs to be validated in other languages and settings.

**Trial registration:**

BlaZIB, NL8106, www.trialregister.nl

**Supplementary Information:**

The online version contains supplementary material available at 10.1186/s12955-022-02064-z.

## Plain English summary

Bladder Cancer is one of the ten most common cancers worldwide. Though, little attention is being paid to the impact of the disease and its treatment on the quality of life of patients. Quality of life can be measured using questionnaires. Multiple organisations developed one or more questionnaires investigating common symptoms and problems of patients presenting with or being treated for bladder cancer. A limitation of most bladder cancer specific questionnaires, in particularly the EORTC-QLQ-BLM30, is the uncertainty about its performance: does it measures what it intends to measure? Can we identify patients with many problems and symptoms? The aim of this study was to answer these questions among others. This study shows that the original grouping of questions of the EORTC-QLQ-BLM30 was inadequate. However, the questionnaire performed well after regrouping of the questions. The results of this study will facilitate urologists and scientists with the interpretation of their patients’ questionnaire data, although some caution is to be remained as only the Dutch version of this questionnaire was examined.

## Introduction

Bladder cancer (BC) is one of the ten most common types of cancer worldwide [1]. About a quarter of the patients presents with Muscle Invasive Bladder Cancer (MIBC) and of three quarter of patients diagnosed with Non-Muscle Invasive Bladder Cancer (NMIBC) 10–15% will subsequently progress to MIBC.

In the past decades the main focus of BC research has been on optimizing oncological outcomes, with relatively little attention being paid to the impact of the disease and its treatment on the functional health, symptom burden and health-related quality of life (HRQoL) of patients [2]. Several reports have indicated that the HRQoL outcomes of patients with BC appear to be worse than those of patients with other cancer types [3]. HRQoL outcomes are typically assessed with patient-reported outcome measures (PROMS). There are currently a number of PROMs designed to assess the HRQoL of patients with BC, including: the Bladder Cancer Index (BCI) [4]; the Functional Assessment of Cancer Therapy questionnaire for bladder cancer patients in general (FACT-Bl) and for those who undergo a cystectomy (FACT-VCI) [5, 6]; and the European Organisation for Research and Treatment of Cancer (EORTC) questionnaires for NMIBC (EORTC-QLQ-NMIBC24) [7, 8] and MIBC (EORTC-QLQ-BLM30) [9]. A major limitation of many of these PROMs is the lack of validation studies demonstrating that these PROMs can accurately measure what they intend to measure [10]. This is especially true for the EORTC-QLQ-BLM30. To date, only one study has investigated the internal consistency of four of seven scales of the QLQ-BLM30 [11] and all other psychometric properties, with the exception of known-group validity [10], have not yet been assessed.

The aim of the current study was to investigate the structural validity, reliability (i.e., internal consistency and test-retest reliability), construct validity (i.e., divergent validity and known group validity), and responsiveness of the Dutch-language version of the EORTC-QLQ-BLM30 in patients with MIBC.

## Methods

### Study design

The study included patients diagnosed with non-metastatic MIBC (≥cT2,cN0–2,cM0) between November 1st 2017 and November 1st 2019, who participated in the HRQoL component of the BlaZIB study (Blaaskankerzorg In Beeld, EN: Insight into bladder cancer care). BlaZIB is a Dutch population-based prospective cohort study, embedded in the Netherlands Cancer Registry (NCR), evaluating the quality of bladder cancer care in the Netherlands. BlaZIB collects comprehensive clinical data and HRQoL data of patients. More information about the BlaZIB study can be found elsewhere [12]. The Committee on Research involving Human Subjects (CMO) of Arnhem-Nijmegen deemed the BlaZIB study exempt from ethical review under the Medical Research Involving Human Subjects Act (WMO). The BlaZIB study was approved by the privacy review board of the NCR. Informed consent, either written or digital, was obtained from all patients participating in the HRQoL component of the BlaZIB study.

### Questionnaires

All patients diagnosed in a hospital participating in the HRQoL component of the BlaZIB study received an invitation to complete the baseline questionnaire shortly, i.e. about 6 weeks, after histological confirmation of the bladder tumour (T6wk). Patients who completed in the baseline questionnaire and were still alive at follow-up received a follow-up questionnaire at 6 months (T6mo), 12 months (T12mo) and 24 months (T24mo) after diagnosis. The questionnaires were provided digitally and in paper-and-pencil format and included questions on demographics, work, lifestyle and HRQoL. HRQoL was assessed with the Dutch version of the EQ-5D-5L, the EORTC-QLQ-C30, the EORTC-QLQ-NMIBC24 and the EORTC-QLQ-BLM30. The Bladder Cancer Index (BCI) was included as an additional non-obligatory questionnaire.

#### Questionnaire scoring

The EORTC-QLQ-C30 consists of 30 items assessing global health status, five functional health domains (physical, role, emotional, cognitive and social functioning) and nine symptoms (fatigue, nausea and vomiting, pain, dyspnoea, insomnia, appetite loss, constipation, diarrhoea and financial difficulties) [13]. The EORTC-QLQ-BLM30 consists of 30 items and originally hypothesized scale to form seven scales (urinary symptom (US), urostomy problem, single catheter use problem (CAT), future worries (FW), abdominal bloating and flatulence (BAF), body image (BI) and sexual functioning) [14]. All items, except for global health status (seven-point scale), are scored on a four-point Likert scale ranging from 1 (not at all) to 4 (very much). Because patients who completed the online questionnaire were required to answer all questions, the response category ‘not applicable / not willing to share’ was added to the items of the sexual functioning scale (items 53 to 60) in the online and paper-and-pencil questionnaire. This extra response category was handled as missing in the calculation of the scores. In accordance with the EORTC guidelines, all responses were linearly transformed to a 0 to 100 scale. and missing data were imputed by averaging the scores of the scale if more than 50% of the items of the scale were completed [15].

#### Additional measures

In order to assess the test-retest reliability of the EORTC-QLQ-BLM30, 81 patients diagnosed with MIBC were asked to complete the EORTC-QLQ-BLM30 2 weeks after the T12mo assessment (T12mo + 2wk; response rate: 84.4%). For practical reasons, this latter questionnaire was only administered in a paper-and-pencil format, but included the same instructions given at T12mo. The T12mo + 2wk questionnaire contained four questions to assess whether patients had less, equal or more complaints in general and on three subscales (urinary, bowel and sexual) compared to the previous questionnaire (T12mo). Only those patients who indicated that they were stable over time on the relevant subscales (i.e. equal complaints) were included in the test-retest analysis.

To assess the divergent validity, the content of the EORTC-QLQ-BLM30 was compared with that of the core questionnaire, the EORTC-QLQ-C30.

### Statistical analyses

#### Structural validity

Confirmatory factor analysis (CFA) was performed to evaluate the hypothesized scale structure of the QLQ-BLM30. Because the US and urostomy problem scales are mutually exclusive, the CFA was run twice, i.e., without US and with urostomy problem and vice versa. Maximum Likelihood (ML) was used as estimator in the CFA and missing items were imputed using Full Info Max Likelihood (fiml). Model-data-fit of the CFA was assessed with model chi-square, the Comparative Fit Index (CFI), Root Mean Square Error of Approximation (RMSEA) and Standardized Root Mean Square Residual (SRMR). Model chi-square > 0.05, CFI ≥0.95, RMSEA < 0.05 and SRMR< 0.05 indicate a good fit, and CFI > 0.90, 0.05 < RMSEA < 0.08 and 0.05 < SRMR < 0.08 indicate an acceptable fit [16].

Multitrait scaling analysis was performed for each assessment point to evaluate the unidimensionality of the scales (i.e., an assumption in classical test theory) and to examine whether the individual items could be grouped in the hypothesized scales. A correlation of ≥0.40 between an item and its own scale was regarded as adequate statistical evidence for convergent validity. Statistical evidence of discriminant validity was defined as a correlation of < 0.40 between an item and other scales in the questionnaire [17]. Items that had poor convergent and/or discriminant validity were discussed and reassigned to another or new scale if necessary. Further psychometric analyses were performed after finalizing the scale structure.

Floor and ceiling effects were examined for each scale. A scale was considered to have a floor or ceiling effect if more than 15% of the patients achieved the lowest or highest possible score, respectively.

#### Measurement error and reliability

Cronbach’s coefficient α was calculated for each scale to assess internal consistency. A Cronbach’s α of 0.70 or higher was considered acceptable for group comparisons. Test-retest reliability was assessed based on the questionnaires administered at T12mo and T12mo + 2wk using the intraclass correlation coefficient (ICC) for absolute agreement (two-way mixed model, single measure) [18]. An ICC value of 0.70 or higher was considered acceptable.

#### Hypothesis testing for construct validity

Divergent validity of the QLQ-BLM30 was assessed by calculating the Spearman correlation coefficients between the scales of the QLQ-C30 and QLQ-BLM30. It was hypothesized that symptoms scales of the QLQ-BLM30 would have low to moderately negative correlations with the functioning scales of the QLQ-C30. A strong correlation was expected between scales that were conceptually related, i.e. constipation and diarrhoea (QLQ-C30) vs abdominal bloating and flatulence (QLQ-BLM30).

Known group validity was assessed by comparing patients with stage II (T2,N0,M0) and stage III (T3-4a,N0,M0 or T1-4A, N1–2, M0) (UICC TNM 2018 [19]) and physical function (PF) < 90 and ≥ 90. It was expected that the HRQoL of patients with stage II and III disease would be comparable as these patients are treated similarly [20]. We hypothesized that patients with high PF (≥90) would report better functioning and less symptoms on all scales than patients with low PF (< 90). Effect sizes (ESs) were calculated using Cohen’s d statistic (mean difference divided by pooled standard deviation). These provide a distribution-based estimate of the magnitude of mean differences/changes, where an ES of 0.2 is considered small, 0.5 moderate, and 0.8 large [21].

#### Responsiveness

Responsiveness to change was assessed in patients who underwent a treatment with curative intent (i.e., radical cystectomy (RC), (chemo) radiotherapy ((C)RT) [20]) after completion of the baseline questionnaire, showed no disease recurrence or progression and completed the EORTC-QLQ-BLM30 questionnaire at all time points. It was hypothesized that patients would report increased urinary, bowel and sexual problems after removal of the bladder compared to baseline [22, 23]. Additionally, we hypothesized that patients who were treated with (C) RT would report better sexual function and body image than patients treated with RC [11].

The CFA was conducted with the software package R using the “lavaan” package [24]. ICCs were calculated in STATA version 16.0 (StataCorp LLC, College Station, Texas, USA). All other statistical analyses were performed using SAS version 9.4 (SAS Institute, Cary, North Carolina, USA).

## Results

### Patient characteristics, completion rates and missing data

**Fig. 1:**
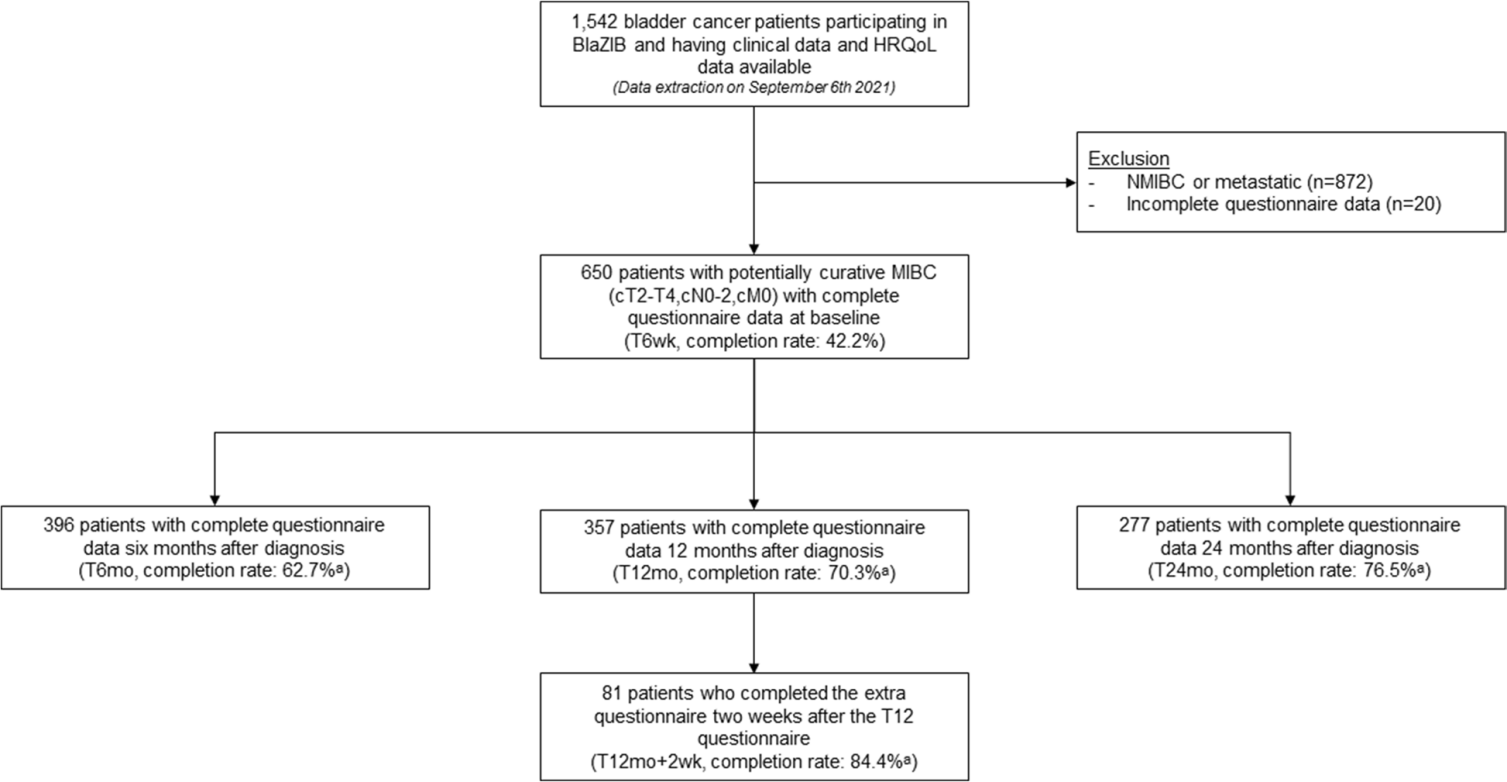
Flowchart. HRQoL = Quality of Life; NMIBC = Non-Muscle Invasive Bladder Cancer; MIBC = Muscle Invasive Bladder Cancer; wk. = week; mo = month. ^a^Percentage of patients that completed the questionnaire after being invited to fill in the questionnaire

Of the 1542 patients invited to participate in the HRQoL measures, 650 patients (42.2%) completed the baseline questionnaire (T6wk). Respondents were more often male, had a slightly better comorbidity profile, had a higher SES, had a more favourable stage distribution and were more likely to undergo a RC (see Additional file 2 for a comparison of the patient and tumour characteristics of the respondents and non-respondents). The follow-up questionnaires had higher completion rates; 396 (62.7% of the invited patients) at T6mo, 357 (70.3%) at T12mo and 277 (76.5%) at T24mo (Fig. 1). The majority of the patients was male (77.7%), living with a partner (76.3%), former smokers (64.8%), and diagnosed with stage II BC (66.2%) (Table 1).

**Table 1:** Patient and tumour characteristics (*n* and %)

	All patients (*n* = 650)	
**Sociodemographic characteristics** (at baseline)		
Gender, no. Male	505	77.7%
Age, mean yr (SD)	72.9	9.8
Age		
< 60	65	10.0%
60- < 70	151	23.2%
70- < 80	265	40.8%
> =80	169	26.0%
Comorbidity (CCI)		
0	280	43.1%
1	186	28.6%
≥ 2	149	22.9%
Unknown	35	5.4%
BMI		
< 23	139	21.4%
23- < 31	445	68.5%
≥ 31	66	10.2%
SES		
Low	152	23.4%
Medium	261	40.2%
High	205	31.5%
Unknown	32	4.9%
Living situation		
With partner	496	76.3%
Without partner	145	22.3%
Else	9	1.4%
Education		
Primary school	92	14.2%
High school	151	23.2%
Secondary vocational education	262	40.3%
Higher professional education	142	21.8%
Unknown	3	0.5%
Employment status		
Paid Job	97	14.9%
No paid job	552	84.9%
Unknown	1	0.2%
Smoking status (at baseline)		
Never	109	16.8%
Former	421	64.8%
Current	144	22.2%
Unknown	6	0.9%
Alcohol use		
Current	471	72.5%
Previous	64	9.8%
Never	109	16.8%
Unknown	6	0.9%
**Clinical characteristics**		
Histology		
Urothelial carcinoma	606	93.2%
Other	44	6.8%
Clinical tumour stage		
cT2, cN0	430	66.2%
cT3, cN0	119	18.3%
cT4a, cN0	22	3.4%
any T, cN1–2	79	12.2%
Treatment		
No treatment	91	14.0%
Cystectomy with neoadjuvant chemotherapy	107	16.5%
Cystectomy alone	214	32.9%
Bladder sparing treatment (chemoradiation, brachytherapy)	104	16.0%
External beam radiotherapy	112	17.2%
Systemic treatment	22	3.4%
Urinary diversion^a^		
Neobladder	21	6.5%
Bricker	296	92.2%
Ureterocutaneous anastomy	2	0.6%
Unknown	2	0.6%

*BMI* Body Mass Index, *CCI *Charlson Comorbidity Index, *SES* Socio-economic status

^a^ Based on the patients treated with a radical cystectomy (*n* = 321)

The percentage of missing responses, including not applicable, on the items single catheter use (item 44) and female sexual function (item 60) were high (> 85%) (see Additional file 2). The percentage of missing responses was low (< 3%) for the items 45 to 52 (future worries, bloating and flatulence and body image scales) and varied per assessment point for the items belonging to the original urinary symptom and urostomy problems scale (items 30 to 43), as these scales are mutually exclusive. The percentage of missing responses for the items belonging to the original sexual function scale (items 53 to 60) was < 48%, except for female sexual function, if limited to the patients reporting at least some sexual activity (item 48).

### Structural validity

Items 44 and 60 were excluded from the CFA because of the high number of missing responses (> 85%). The hypothesized scale structure of the QLQ-BLM30 did not fit the data well, with: a CFI of 0.80–0.86, RMSEA of 0.07–0.08 and SRMR of 0.11–0.13 (see Additional file 2). Multitrait scaling analysis showed that the sexual functioning and urostomy problems scales were particularly problematic (see Additional file 2). For this reason, we decided to revise the sexual functioning scale in the same way as was done for the EORTC-QLQ-NMIBC24 [7] (see Table 3), even though items 57 and 58 showed a moderate correlation (0.53–0.59) and the model fit remained largely the same (±0.002 change) after combining these items into one scale (Additional file 2).

Based on the data and the item content, the urostomy problems scale was reduced to a three-item scale (items 38, 39 and 43). The remaining items in the originally hypothesized scale about urostomy irritation (item 40), urostomy embarrassment (item 41) and urostomy support (item 42) were kept as single items. Although the bloating and flatulence scale showed low convergent validity (< 0.40) at all time points (see Table 2), it was considered to be an unidimensional scale in patients who underwent a RC (con: 0.43; dis: − 0.07 to 0.31). This led to the decision to keep the bloating and flatulence scale intact. The revised scale structure exhibited adequate to good fit at all time points (see Additional file 2).

**Table 2:** Item correlations within and between scales of the EORTC QLQ-BLM30 at baseline (T6wk), 6 months, 12 months and 24 months

Scale	Baseline (n=650)									6-mo follow-up (n=396)									12-mo follow-up (n=357)									24-mo follow-up (n=277)								
	Con			Dis			α	%Floor	%Ceiling	Con			Dis			α	%Floor	%Ceiling	Con			Dis			α	%Floor	%Ceiling	Con			Dis			α	%Floor	%Ceiling
US^a^	0.51 to 0.72			-0.17 to 0.29			0.87	42.7%	0.9%	0.55 to 0.77			-0.19 to 0.38			0.89	45.0%	0.5%	0.64 to 0.77			-0.26 to 0.44			0.90	50.0%	0.0%	0.41 to 0.78			-0.51 to 0.59			0.87	48.3%	0.0%
UP^b^	0.34 to 0.39			-0.16 to 0.33			0.58	53.1%	0.8%	0.33 to 0.39			-0.17 to 0.19			0.60	40.3%	0.8%	0.41 to 0.46			-0.19 to 0.24			0.65	45.6%	0.6%	0.21 to 0.49			-0.11 to 0.31			0.55	44.6%	0.4%
FW	0.77 to 0.82			-0.03 to 0.38			0.89	7.8%	5.2%	0.75 to 0.82			-0.17 to 0.38			0.89	21.4%	1.0%	0.70 to 0.80			-0.35 to 0.45			0.87	26.7%	0.3%	0.79 to 0.82			-0.22 to 0.48			0.90	35.8%	0.8%
BAF	0.37 to 0.37			-0.12 to 0.28			0.54	55.6%	0.0%	0.37 to 0.37			-0.08 to 0.34			0.54	51.6%	0.0%	0.37 to 0.37			-0.17 to 0.36			0.54	46.0%	0.4%	0.32 to 0.32			-0.16 to 0.54			0.48	46.1%	0.0%
BI	0.54 to 0.67			-0.08 to 0.42			0.78	39.8%	17.0%	0.57 to 0.70			-0.08 to 0.31			0.78	30.5%	30.5%	0.61 to 0.70			-0.18 to 0.44			0.80	33.3%	25.8%	0.60 to 0.69			-0.11 to 0.47			0.80	31.1%	27.7%
SX	0.70 to 0.70			-0.17 to 0.03			0.82	11.4%	0.0%	0.60 to 0.60			-0.20 to -0.01			0.75	39.6%	0.5%	0.64 to 0.64			-0.31 to 0.04			0.78	51.0%	0.0%	0.66 to 0.66			-0.46 to 0.00			0.79	55.5%	0.0%
SXmen	0.77 to 0.77			-0.16 to 0.18			0.87	7.1%	0.4%	0.64 to 0.64			-0.12 to 0.27			0.78	6.8%	0.0%	0.65 to 0.65			-0.13 to 0.27			0.79	12.7%	0.6%	0.70 to 0.70			-0.16 to 0.21			0.82	17.8%	0.0%

α Cronbach α coefficient, *BAF* Bloating and flatulence, *BI* Body image, *Con* Range of item-scale correlations (corrected for overlap), *Dis* Range of correlations between an item and another scale, *FW* Future worries, *SX* Sexual functioning, *SXmen* Sexual problems in men, *UP* Urostomyproblems, *US* Urinary symptoms

^a^ Results are based on 546 (T6wk), 206 (T6mo), 158 (T12mo) and 107 (T24mo) patients

^b^ Results are based on 88 (T6wk), 182 (T6mo), 192 (T12mo) and 164 (T24mo) patients

The hypothesized and revised scale structures of the EORTC-QLQ-BLM30 are shown in Table 3.

**Table 3:** The originally hypothesized and revised scale structure of the EORTC QLQ-BLM30

Originally hypothesized scales	Items	Revised scales and single items	Items
Urinary symptom	31–37	Urinary symptom (US)	31–37
Urostomy problem	38–43	Urostomy problem (UP)	38,39,43
Single cathether use problem	44	Single catheter use problem (CAT)	44
Future worries	45–47	Future worries (FW)	45–47
Abdominal bloating and flatulence	48–49	Abdominal bloating and flatulence (BAF)	48–49
Body image	50–52	Body image (BI)	50–52
Sexual functioning	53–60	Sexual functioning (SX)	53–54
		Male sexual problems (SXmen)	55–56
		Urostomy irritation (UPi)	40
		Urostomy embarrassment (UPe)	41
		Urostomy support (UPs)	42
		Sexual intimacy (SXI)	57
		Risk of contaminating partner (SXcp)	58
		Sexual enjoyment (SXen)	59
		Female sexual problems (SXfem)	60

### Measurement error and reliability

The internal consistency of the revised scales at all time points was good (α > 0.70), with the exception of urostomy problems (0.55–0.65) and bloating and flatulence (0.48–0.54; Table 2). Test-retest reliability was acceptable for three scales (ICC > 0.70); nearly acceptable for two scales (ICC 0.68–0.69) and fair to moderate for the urostomy problems scale (0.61) and bloating and flatulence (0.47; Table 4).

**Table 4:** Interclass correlation coefficient of the QLQ-BLM30 subscales for 81 MIBC patients participating in the test-retest analysis of BlaZIB

Scale	Assessment of stability	Number of stable patients^a^	ICC	(95% CI)	SEM^b^
*Scales*					
US	urinary function	29	0.76	(0.55–0.88)	8.9
UP	urinary function	24	0.61	(0.29–0.81)	10.5
FW	total function	42	0.68	(0.48–0.81)	13.7
BAF	bowel function	39	0.47	(0.18–0.68)	11.0
BI	total function	40	0.80	(0.64–0.89)	8.9
SX	sexual function	36	0.69	(0.50–0.82)	12.1
SXmen	sexual function	29	0.73	(0.53–0.86)	20.8
*Single items*					
CAT	urinary function	2	*NA*	*NA*	*NA*
UPi	urinary function	24	0.79	(0.56–0.90)	9.6
UPe	urinary function	25	0.57	(0.24–0.79)	10.5
UPs	urinary function	24	0.76	(0.51–0.89)	11.8
SXi	sexual function	17	0.42	(−0.03–0.73)	17.0
SXcp	sexual function	16	0.21	(−0.27–0.60)	24.2
SXen	sexual function	17	0.74	(0.42–0.90)	16.1
SXfem	sexual function	0	*NA*	*NA*	*NA*

*BAF* Abdominal bloating and flatulence, *BI* Body image, *CAT* Single catheter use problem, *CI* Confidence Interval, *FW* Future worries, *ICC* Intraclass Correlation Coefficient, *UP* Urostomy problem, *UPe* Urostomy embarrassment, UPi Urostomy irritation, *Ups* Urostomy support, *US* Urinary symptom, *SEM* Standard Error of Measure, *SX* Sexual functioning, *SXcp* Risk of contaminating partner, *SXen* Sexual enjoyment, *SXfem* Female sexual problems, *SXi* Sexual intimacy, *SXmen* Male sexual problems

^a^ Patients who (self-reported) remained the same with respect to the specific assessment of stability (i.e. urinary, bowel, sexual or total function) between the T12mo and T12mo + 2wk questionnaire

^b^ Using the Restricted Maximum Likelihood (REML) approach

### Hypothesis testing for construct validity

The correlations between the scales of the core questionnaire (QLQ-C30) and QLQ-BLM30 questionnaire were low (< 0.40; Table 5), with the exception of the correlation between emotional function (QLQ-C30) and future worries (QLQ-BLM30). This indicates that the module’s content does not, for the most part, overlap with the content of the core questionnaire.

**Table 5:** Correlations between scales of the QLQ-C30 and QLQ-BLM30 at baseline

QLQ-C30 scales	QLQ-BLM30						
	US^b^	UP^b^	FW^b^	BAF^b^	BI^b^	SX^a^	SXmen^b^
Global health status / QoL^a^	− 0.28	− 0.33	− 0.35	− 0.22	− 0.33	0.23	− 0.10
*Functioning scales*^*a*^							
Physical function	−0.26	− 0.38	− 0.16	− 0.24	− 0.32	0.37	− 0.17
Role function	− 0.23	− 0.24	− 0.22	− 0.27	−0.33	0.21	−0.10
Emotional function	−0.15	− 0.30	− 0.60	− 0.26	− 0.33	0.09	− 0.02
Cognitive function	−0.16	− 0.24	−0.24	− 0.24	− 0.24	0.12	−0.14
Social function	−0.25	−0.41	− 0.30	−0.24	− 0.38	0.20	− 0.14
*Symptom scales*^*b*^							
Nausea and vomiting	0.29	0.35	0.28	0.29	0.34	−0.24	0.20
Fatigue	0.16	0.13	0.14	0.18	0.13	−0.07	0.05
Pain	0.34	0.03	0.24	0.28	0.29	−0.17	0.03

*BAF* Bloating and flatulence, *BI* Body image, *FW* Future worries, *SX* Sexual functioning, *SXmen* Sexual problems in men, *UP* Urostomy problems, *US* Urinary symptoms, *Q0L* Quality of Life

^a^ A higher score on this scale indicates better functioning

^b^ A higher score on this scale indicates more symptoms, problems or worries

The scores of patients with stage II and stage III bladder cancer were, as expected, quite similar (ES < 0.30). Patients with a score of 0.90 or higher on the physical function scale of the QLQ-C30 had higher scores on the functional scales and lower scores on the symptom scales of the QLQ-C30 and QLQ-BLM30 compared to patients with physical function scores < 0.90 (Table 6).

**Table 6:** Comparison of mean scores for the QLQ-C30 and QLQ-BLM30 between patients with stage II and stage III bladder cancer and with high and low physical function at baseline (T6wk)

	Total		Stage II (*n* = 430)		Stage III (*n* = 220)		Effect size^c^	PF > =90 (*n* = 228)		PF < 90 (*n* = 422)		Effect size^c^
	Mean	SD	Mean	SD	Mean	SD		Mean	SD	Mean	SD	
QLQ-C30												
Global health status / QoL^a^	69.4	19.5	70.6	18.9	66.9	20.3	0.19	81.1	14.1	62.9	19.0	0.93
*Functioning scales*^*a*^												
Physical function	76.6	22.6	76.1	23.1	77.5	21.6	−0.06	97.2	3.3	65.3	20.6	1.41
Role function	72.2	29.2	73.0	29.0	70.8	29.5	0.08	90.6	17.8	62.2	29.3	0.97
Emotional function	79.9	19.9	80.5	20.3	78.8	19.1	0.08	85.3	14.5	77.0	21.7	0.42
Cognitive function	86.6	18.7	86.9	17.9	85.8	20.1	0.06	94.0	10.8	82.5	20.7	0.62
Social function	81.5	23.5	81.6	23.7	81.3	23.0	0.01	91.7	15.1	75.8	25.3	0.68
*Symptom scales*^*b*^												
Fatigue	32.0	25.3	31.3	24.3	33.3	27.1	−0.08	16.3	18.7	40.6	24.3	−0.96
Nausea and vomiting	6.2	14.8	5.8	13.9	7.2	16.4	−0.09	2.6	8.3	8.3	17.1	−0.38
Pain	20.4	24.2	18.6	22.5	23.9	26.9	−0.22	8.7	15.8	26.8	25.6	−0.75
QLQ-BLM30												
*Scales*												
Urinary symptom^b^	34.1	22.7	32.5	21.9	37.5	23.9	−0.04	28.7	21.6	37.6	22.7	−0.39
Urostomy problem^b^	28.3	21.4	27.9	22.3	28.8	20.3	−0.11	18.9	14.9	29.5	21.8	−0.50
Future perspective^b^	43.4	25.9	42.4	25.2	45.4	27.2	−0.05	40.1	23.6	45.3	27.0	−0.20
Abdominal bloating and flatulence^b^	17.4	20.2	17.1	20.4	18.0	19.7	−0.08	11.9	16.5	20.4	21.3	−0.42
Body image^b^	13.9	19.8	13.3	18.9	14.9	21.5	0.04	7.5	14.7	17.3	21.4	−0.49
Sexual functioning^a^	14.9	19.8	15.2	20.2	14.4	19.0	0.10	23.3	21.0	10.0	17.2	0.68
Male sexual problems^b^	34.5	37.4	35.8	38.3	31.9	35.5	0.05	25.7	29.1	41.2	41.5	−0.41
*Single items*												
Single cathether use problem	32.5	37.8	33.3	38.7	31.4	37.0	0.05	11.8	20.2	37.7	39.4	−0.69
Urostomy irritation^b^	23.5	25.8	20.3	25.9	27.9	25.5	−0.30	20.0	23.3	23.9	26.3	−0.15
Urostomy embarrassment^b^	24.1	32.0	24.7	33.5	23.4	30.3	0.04	13.3	17.2	25.5	33.3	−0.38
Urostomy support^b^	55.3	34.2	55.6	35.1	55.0	33.5	0.02	40.0	30.6	57.3	34.4	−0.50
Sexual intimacy^b^	15.9	26.7	15.6	26.6	16.4	27.0	−0.03	12.9	23.5	18.3	28.8	−0.20
Risk of contaminating partner^b^	16.0	28.3	14.7	27.4	18.6	30.0	−0.14	13.9	26.6	17.9	29.7	−0.14
Sexual enjoyment^a^	29.6	34.3	28.3	33.3	32.4	36.1	−0.12	40.0	34.4	20.9	31.7	0.56
Female sexual problems^b^	20.7	28.7	20.5	28.4	21.1	29.8	−0.02	20.7	28.7	8.3	15.1	−0.59

*PF* Physical functioning, *SD* Standard Deviation, *QoL* Quality of Life

^a^ A higher score on this scale indicates better functioning

^b^ A higher score on this scale indicates more symptoms, problems or worries

^c^ Effect size is mean difference divided by the standard deviation of the total population

### Responsiveness

Future worries decreased after baseline in patients who underwent treatment with curative intent (EF = 0.67 to 1.39; Table 7). Body image (EF= –0.77 to –0.62) and male sexual problems (EF= –0.78 to –0.67) deteriorated in patients who underwent a RC, RC, while body image (EF = 0.23 to 0.33) and urinary function (EF = 0.16 to 0.59) improved in patients undergoing (C)RT.

**Table 7:** Responsiveness to change over time in patients who underwent a potential curative therapy, completed the EORTC-QLQ-BLM30 at all time points and had no disease recurrence or progression

	T6wk		T6mo			T12mo			T24mo		
	mean	(SD)	mean	(SD)	Effect size^c^	mean	(SD)	Effect size^c^	mean	(SD)	Effect size^c^
RC patients (*n* = 101)											
US^a^	30.3	20.7	.	.	.	.	.		.	.	
UP^a^	.	.	15.1	17.7	.	9.8	12.6	0.35^d^	8.6	12.8	0.42^d^
FW^a^	47.2	23.0	24.3	20.8	1.05	19.9	16.4	1.25	16.9	18.3	1.39
BAF^a^	13.7	18.0	13.8	16.9	−0.01	11.6	15.3	0.12	13.0	14.6	0.04
BI^a^	9.2	13.1	23.0	21.7	−0.77	20.4	20.9	−0.63	20.3	19.4	−0.62
SX^b^	22.1	20.7	17.4	19.1	0.24	20.8	20.3	0.07	21.4	20.8	0.03
Sxmen^a^	27.2	27.3	50.8	40.7	−0.68	54.3	40.0	−0.78	50.5	41.7	−0.67
(C) RT patients (*n* = 56)											
US^a^	30.9	18.2	27.6	23.0	0.16	19.6	19.8	0.59	24.5	21.7	0.32
FW^a^	45.0	26.6	27.5	26.0	0.67	21.8	20.4	0.98	19.1	22.2	1.06
BAF^a^	13.1	18.5	13.9	17.7	−0.04	13.9	19.9	−0.04	13.0	15.1	0.01
BI^a^	12.5	20.3	7.0	11.7	0.33	7.0	15.0	0.31	8.4	15.4	0.23
SX^b^	19.2	23.3	16.7	22.0	0.11	19.6	26.4	−0.02	20.8	22.7	−0.07
Sxmen^a^	23.7	35.0	29.2	34.1	−0.16	19.6	28.0	0.13	37.6	55.8	−0.30

*BAF* Bloating and flatulence, *BI* Body image, *(C) RT* (Chemo) radiotherapy, *FW* Future worries, *mo* Month, *RC* Radical Cystectomy, *SD* Standard deviation, *SX* Sexual functioning, *SXmen* Sexual problems in men, *UP* Urostomy problems, *US* Urinary symptoms; wk. = week

^a^ A higher score on this scale indicates more symptoms, problems or worries

^b^ A higher score on this scale indicates better functioning

^c^ Effect size is mean difference (i.e. between T6wk and time point unless otherwise indicated) divided by the pooled standard deviation

^d^ Effect size is mean difference (i.e. between T6mo and time point) divided by the pooled standard deviation

## Discussion

The aim of this study was to examine the structural validity, reliability, construct validity and responsiveness of the Dutch version of the EORTC-QLQ-BLM30. The original hypothesized scale structure of the QLQ-BLM30 could not be substantiated using data of 650 Dutch patients with MIBC, and therefore, the scale structure was revised into seven scales (urinary symptoms, urostomy problems, future worries, abdominal bloating and flatulence, body image, sexual functioning and male sexual problems) and eight single items. The revised scale structure, in general, exhibited good reliability, construct validity and responsiveness. Only reliability (i.e. internal consistency and test-retest reliability) of the new urostomy problems scale and abdominal bloating and flatulence scale was below the acceptable cut-off point.

Based on the data and the items’ content, we revised the six-item urostomy problems scale into a three-item scale and three single items. The new urostomy problem scale performs better (i.e., has higher internal consistency and better CFA results) than the originally hypothesized scale, but still performs below the acceptable cut-off point for both internal consistency and test-retest reliability. We would note that the original hypothesized scale showed better internal consistency (α = 0.71) in the study of Mak et al. [11]. This indicates that more research will be needed to examine the coherence of the items 38 to 43, as the possibility exists that the originally hypothesized urostomy problem scale may be sufficient in other populations than the current study population.

The two items of the abdominal bloating and flatulence scale (i.e., items 48 and 49) were only moderately correlated and thus appeared to measure one unidimensional construct in the general MIBC population. Furthermore, the test-retest analysis indicated that the scores on this scale varied more than would be desirable in generally stable patients, and no large differences were observed for this scale in patients who experienced changes in health over time. Similar results have been observed for this scale in patients diagnosed with NMIBC [7, 8]. However, the correlation between the items of the bloating and flatulence scale was higher for patients treated with RC compared to the total MIBC population. It may be that the bloating and flatulence scale is not relevant for the entire MIBC population, but only for certain subgroups such as patients treated with RC or (C)RT. More research is needed to explore this scale further in other populations and patient subgroups.

The response rates for the items addressing sexual function, especially female sexual function (item 60), were generally lower than for the items of other scales if corrected for the missing responses related to having and not having had a urostomy (i.e., urinary symptom and urostomy problem scale). This finding is in line with other studies [7, 22, 25], but nevertheless resulted in the exclusion of female sexual function and the item single catheter use from the CFA. The new grouping of the items addressing sexual function is the same as proposed and confirmed for the QLQ-NMIBC24 [7, 8, 25], and therefore, we expect that this new grouping of the sexual items will be sustained in future studies investigating the measurement properties of the QLQ-BLM30.

Although this study evaluated the measurement properties of the QLQ-BLM30 in a large population-based group of patients with MIBC, it has some limitations. The primary limitation is its setting; this study was performed in a single study and country. Further research will be needed to examine the measurement properties of the QLQ-BLM30 in other countries and settings. Furthermore, the completion rate of the baseline questionnaire (T6wk) was rather low, which may affect the generalizability of the scores to the entire Dutch population with MIBC. We do, however, believe that this would have a negligible effect on the observed measurement properties of the QLQ-BLM30 as it is unlikely that potential selection bias significantly affect the measurement properties of the questionnaire. Finally, we would note that we relied on classical psychometrics to evaluate the properties of the QLQ-BLM30. Clinimetric evaluation of the questionnaire might result in a somewhat different scale structure than reported here, because that approach does not require unidimensionality and homogeneity of components [26]. Clinimetric evaluation may additionally provide insight into the clinical utility and sensitivity of the questionnaire.

## Conclusion

The originally hypothesized scale structure of the EORTC-QLQ-BLM30 did not fit the data well and needed revision. The proposed revised scale structure of the QLQ-BLM30, in general, exhibits good structural validity, reliability (i.e., internal consistency and test-retest reliability), construct validity (i.e., divergent validity and known group validity) and responsiveness in Dutch patients with MIBC. The urostomy problem and bloating and flatulence scale properties remain suboptimal and further studies are needed to confirm our findings.

## Supplementary Information

**Additional file 1.**

**Additional file 2.**

## Data Availability

The datasets generated during and/or analysed during the current study are available from the corresponding author on reasonable request.
