# Supplementary material for: Validation and reliability of the Dutch version of the EORTC QLQ-BLM30 module for assessing the health-related quality of life of patients with muscle invasive bladder cancer
Source: Health Qual Life Outcomes. 2022 Dec 29;20:171. doi: 10.1186/s12955-022-02064-z (PMC9798594; doi:10.1186/s12955-022-02064-z)
Supplement: Supplementary file 1 — Additional file 1. [file 12955_2022_2064_MOESM1_ESM.pdf]

Additional file 1

Table A.1 Patient and tumour characteristics of responders and non-respondents at baseline

	Responders (n=650)		Non-respondents[a] (n=892)		P-value
**Sociodemographic characteristics** (at baseline)					
Gender, no. Male	505	77.7%	591	66.3%	<0.0001
Age, mean yr (SD)	72.9	(9.8)	72.5	(10.7)	0.38
Age					
<60	65	10.0%	116	13.0%	0.29
60-<70	151	23.2%	196	22.0%	
70-<80	265	40.8%	342	38.3%	
>=80	169	26.0%	238	26.7%	
Comorbidity (CCI)					
0	280	43.1%	330	37.0%	0.04
1	186	28.6%	298	33.4%	
2	149	22.9%	217	24.3%	
>2	35	5.4%	47	5.3%	
SES					
Low	152	23.4%	313	35.1%	<0.0001
Medium	261	40.2%	316	35.4%	
High	205	31.5%	207	23.2%	
Unknown	32	4.9%	56	6.3%	
**Clinical characteristics**					
Histology					0.18
Urothelial cell carcinoma	606	93.2%	846	94.8%	
Else	44	6.8%	46	5.2%	
Clinical tumour stage, no.					0.05
cT2, cN0	430	66.2%	537	60.2%	
cT3, cN0	119	18.3%	199	22.3%	
cT4a, cN0	22	3.4%	47	5.3%	
any T, cN1-2	79	12.2%	109	12.2%	
Treatment					<0.0001
No treatment	91	14.0%	220	24.7%	
Cystectomy with NAC	107	16.5%	139	15.6%	
Cystectomy alone	214	32.9%	234	26.2%	
Bladder sparing treatment (chemoradiation, brachytherapy)	104	16.0%	96	10.8%	
EBRT	112	17.2%	166	18.6%	
Systemic treatment	22	3.4%	37	4.1%	
Urinary diversion[b]					0.28
Neobladder	21	6.5%	14	1.6%	
Indiana pouch	0	0.0%	1	0.1%	
Bricker	296	92.2%	349	39.1%	
Ureterocutaneous anostomy	2	0.6%	6	0.7%	
Unknown	2	0.6%	3	0.3%	

CCI = Charlson Comorbidity Index; SES = Socio-economic status\

[a] Non-respondents and respondents who did not complete the first questionnaire (n=29).

[b] Based on the patients treated with a radical cystectomy for respondents (n=321) and non-respondents (n=373)
