# Supplementary material for: Validation and reliability of the Dutch version of the EORTC QLQ-BLM30 module for assessing the health-related quality of life of patients with muscle invasive bladder cancer
Source: Health Qual Life Outcomes. 2022 Dec 29;20:171. doi: 10.1186/s12955-022-02064-z (PMC9798594; doi:10.1186/s12955-022-02064-z)
Supplement: Supplementary file 2 — Additional file 2. [file 12955_2022_2064_MOESM2_ESM.pdf]

Additional file 2

Table B.1 Fit statistics of the confirmatory factor analysis[a]

	CFI	TLI	RMSEA	SRMR
**T6wk**				
Model with US				
Original scale structure	0.839	0.813	0.079	0.106
Revision SX scale 1 (=revised scale)	0.949	0.933	0.047	0.041
Revision SX scale 2: 57+58	0.947	0.932	0.048	0.043
Model with UP				
Original scale structure	0.802	0.767	0.076	0.133
Revision SX scale 1	0.960	0.950	0.034	0.090
Revision UP scale (=revised scale)	0.970	0.951	0.035	0.067
Revision SX scale 2	0.969	0.953	0.034	0.068
**T6mo**				
Model with US				
Original scale structure	0.857	0.834	0.067	0.106
Revision SX scale 1 (=revised scale)	0.943	0.925	0.045	0.059
Revision SX scale 2: 57+58	0.944	0.929	0.044	0.062
Model with UP				
Original scale structure	0.824	0.793	0.069	0.104
Revision SX scale 1	0.934	0.912	0.045	0.073
Revision UP scale (=revised scale)	0.954	0.926	0.041	0.049
Revision SX scale 2	0.955	0.932	0.039	0.053
**T12mo**				
Model with US				
Original scale structure	0.807	0.776	0.078	0.112
Revision SX scale 1 (=revised scale)	0.920	0.895	0.053	0.133
Revision SX scale 2: 57+58	0.920	0.900	0.052	0.143
Model with UP				
Original scale structure	0.781	0.743	0.079	0.113
Revision SX scale 1	0.923	0.896	0.051	0.076
Revision UP scale (=revised scale)	0.935	0.895	0.051	0.069
Revision SX scale 2	0.933	0.899	0.050	0.068

CFI = Comparative Fit Index; mo = month; UP = Urostomy problems; RMSEA = Root Mean Square Error of Approximation; SRMR = Standardized Root Mean Square Residual; SX = Sexual functioning; TLI = Tucker-Lewis Index; wk = week

[a] The items 44 and 60 were not included in the confirmatory factor analysis due to the high number of missing responses.

Table B.2 Number of missing responses, floor and ceiling effects, convergent and discriminant validity of the original scale structure of the EORTC-QLQ-BLM30 at T6wk (N=650)

Item	Hypothesized scale	Missings		Floor		Ceiling		Convergent validity	Divergent validity
		N	(%)	N	(%)[a]	N	(%)[a]		
Q31	US	104	16.0%	84	15.4%	63	11.5%	0.66	0.01 to 0.15
Q32	US	107	16.5%	67	12.3%	84	15.5%	0.72	-0.01 to 0.19
Q33	US	107	16.5%	167	30.8%	68	12.5%	0.72	0.05 to 0.19
Q34	US	110	16.9%	242	44.8%	42	7.8%	0.70	0.03 to 0.21
Q35	US	106	16.3%	297	54.6%	22	4.0%	0.69	0.03 to 0.29
Q36	US	109	16.8%	345	63.8%	17	3.1%	0.55	0.08 to 0.24
Q37	US	107	16.5%	265	48.8%	38	7.0%	0.51	0.03 to 0.26
Q38[b]	UP	598	92.0%	29	55.8%	3	5.8%	0.36	-0.06 to 0.25
Q39[b]	UP	600	92.3%	21	42.0%	7	14.0%	0.64	0.07 to 0.48
Q40[b]	UP	598	92.0%	19	36.5%	2	3.8%	0.45	0.00 to 0.26
Q41[b]	UP	599	92.2%	29	56.9%	5	9.8%	0.50	0.02 to 0.43
Q42[b]	UP	598	92.0%	6	11.5%	17	32.7%	0.48	-0.07 to 0.29
Q43[b]	UP	599	92.2%	17	33.3%	5	9.8%	0.35	0.13 to 0.43
Q44	CAT	565	86.9%	44	51.8%	11	12.9%		-0.04 to 0.46
Q45	FP	11	1.7%	83	13.0%	50	7.8%	0.77	0.08 to 0.38
Q46	FP	14	2.2%	115	18.1%	58	9.1%	0.82	0.09 to 0.36
Q47	FP	15	2.3%	128	20.2%	67	10.6%	0.78	0.10 to 0.36
Q48	BAF	14	2.2%	444	69.8%	12	1.9%	0.37	0.01 to 0.28
Q49	BAF	15	2.3%	324	51.0%	9	1.4%	0.37	0.08 to 0.21
Q50	BI	16	2.5%	452	71.3%	11	1.7%	0.64	0.18 to 0.46
Q51	BI	16	2.5%	421	66.4%	16	2.5%	0.54	0.08 to 0.35
Q52	BI	17	2.6%	455	71.9%	8	1.3%	0.67	0.17 to 0.32
Q53	SX	108	16.6%	326	60.1%	2	0.4%	0.23	-0.17 to -0.04
Q54	SX	113	17.4%	358	66.7%	1	0.2%	0.27	-0.15 to 0.03
Q55	SX	268	41.2%	171	44.8%	71	18.6%	0.39	-0.01 to 0.11
Q56	SX	313	48.2%	184	54.6%	61	18.1%	0.34	0.08 to 0.17
Q57	SX	306	47.1%	234	68.0%	13	3.8%	0.30	0.03 to 0.51
Q58	SX	319	49.1%	235	71.0%	15	4.5%	0.31	-0.01 to 0.27
Q59	SX	334	51.4%	157	49.7%	29	9.2%	0.22	-0.35 to 0.00
Q60	SX	605	93.1%	27	60.0%	1	2.2%	0.56	-0.28 to 0.37

[a] Percentage of the patients with non-missing data

[b] Only a small number of patients had undergone a radical cystectomy at T6wk, and therefore, the scores of the UP scale should be interpreted with some caution.

Table B.3 Number of missing responses, floor and ceiling effects, convergent and discriminant validity of the original scale structure of the EORTC-QLQ-BLM30 at T6mo (N=396)

Item	Hypothesized scale	Missings		Floor		Ceiling		Convergent validity	Divergent validity		
		N	(%)	N	(%)[a]	N	(%)[a]				
Q31	US	202	51.0%	31	16.0%	23	11.9%	0.72	-0.02	to	0.31
Q32	US	204	51.5%	25	13.0%	33	17.2%	0.75	0.10	to	0.37
Q33	US	204	51.5%	71	37.0%	27	14.1%	0.76	0.04	to	0.33
Q34	US	203	51.3%	87	45.1%	15	7.8%	0.71	0.19	to	0.36
Q35	US	202	51.0%	103	53.1%	11	5.7%	0.74	0.21	to	0.36
Q36	US	202	51.0%	118	60.8%	7	3.6%	0.53	0.11	to	0.37
Q37	US	202	51.0%	120	61.9%	7	3.6%	0.55	0.01	to	0.62
Q38	UP	227	57.3%	116	68.6%	3	1.8%	0.44	-0.18	to	0.25
Q39	UP	229	57.8%	130	77.8%	3	1.8%	0.49	-0.07	to	0.38
Q40	UP	226	57.1%	83	48.8%	6	3.5%	0.14	-0.09	to	0.13
Q41	UP	226	57.1%	120	70.6%	1	0.6%	0.14	-0.13	to	0.42
Q42	UP	226	57.1%	89	52.4%	14	8.2%	0.39	-0.04	to	0.34
Q43	UP	232	58.6%	81	49.4%	1	0.6%	0.58	-0.13	to	0.23
Q44	CAT	369	93.2%	18	66.7%	1	3.7%		-0.10	to	0.37
Q45	FP	8	2.0%	102	26.3%	13	3.4%	0.75	-0.07	to	0.35
Q46	FP	8	2.0%	134	34.5%	9	2.3%	0.82	-0.10	to	0.44
Q47	FP	12	3.0%	151	39.3%	9	2.3%	0.79	-0.09	to	0.38
Q48	BAF	9	2.3%	297	76.7%	2	0.5%	0.37	0.08	to	0.49
Q49	BAF	16	4.0%	185	48.7%	8	2.1%	0.37	0.04	to	0.28
Q50	BI	12	3.0%	242	63.0%	8	2.1%	0.70	0.04	to	0.30
Q51	BI	13	3.3%	230	60.1%	7	1.8%	0.57	0.10	to	0.45
Q52	BI	12	3.0%	211	54.9%	11	2.9%	0.60	0.11	to	0.29
Q53	SX	91	23.0%	164	53.8%	4	1.3%	0.12	-0.22	to	-0.01
Q54	SX	93	23.5%	205	67.7%	0	0.0%	0.11	-0.21	to	-0.03
Q55	SX	173	43.7%	86	38.6%	81	36.3%	0.43	-0.19	to	0.27
Q56	SX	201	50.8%	69	35.4%	77	39.5%	0.40	-0.10	to	0.25
Q57	SX	220	55.6%	107	60.8%	7	4.0%	0.22	-0.19	to	0.36
Q58	SX	227	57.3%	120	71.0%	9	5.3%	0.26	-0.14	to	0.27
Q59	SX	229	57.8%	74	44.3%	11	6.6%	0.14	-0.24	to	0.37
Q60	SX	381	96.2%	10	66.7%	1	6.7%	0.22	-0.24	to	0.58

[a] Percentage of the patients with non-missing data

Table B.4 Number of missing responses, floor and ceiling effects, convergent and discriminant validity of the original scale structure of the EORTC-QLQ-BLM30 at T12mo (N=357)

Item	Hypothesized scale	Missings		Floor		Ceiling		Convergent validity	Divergent validity		
		N	(%)	N	(%)[a]	N	(%)[a]				
Q31	US	214	59.9%	38	26.6%	12	8.4%	0.68	-0.36	to	0.35
Q32	US	213	59.7%	28	19.4%	16	11.1%	0.75	-0.40	to	0.33
Q33	US	212	59.4%	64	44.1%	14	9.7%	0.67	-0.31	to	0.44
Q34	US	216	60.5%	80	56.7%	6	4.3%	0.65	-0.31	to	0.37
Q35	US	217	60.8%	90	64.3%	7	5.0%	0.77	-0.21	to	0.39
Q36	US	214	59.9%	91	63.6%	8	5.6%	0.71	-0.24	to	0.39
Q37	US	214	59.9%	95	66.4%	7	4.9%	0.64	-0.13	to	0.38
Q38	UP	176	49.3%	132	72.9%	1	0.6%	0.41	-0.03	to	0.24
Q39	UP	176	49.3%	155	85.6%	2	1.1%	0.54	-0.07	to	0.16
Q40	UP	175	49.0%	97	53.3%	0	0.0%	0.31	0.02	to	0.09
Q41	UP	175	49.0%	139	76.4%	0	0.0%	0.34	0.11	to	0.31
Q42	UP	174	48.7%	124	67.8%	12	6.6%	0.31	0.02	to	0.22
Q43	UP	175	49.0%	112	61.5%	4	2.2%	0.53	0.05	to	0.20
Q44	CAT	333	93.3%	18	75.0%	2	8.3%		-0.30	to	0.13
Q45	FP	13	3.6%	116	33.7%	9	2.6%	0.70	-0.12	to	0.45
Q46	FP	14	3.9%	135	39.4%	5	1.5%	0.80	-0.04	to	0.40
Q47	FP	14	3.9%	168	49.0%	5	1.5%	0.76	-0.08	to	0.33
Q48	BAF	16	4.5%	271	79.5%	1	0.3%	0.37	0.00	to	0.36
Q49	BAF	18	5.0%	184	54.3%	6	1.8%	0.37	-0.03	to	0.23
Q50	BI	16	4.5%	220	64.5%	6	1.8%	0.70	-0.11	to	0.36
Q51	BI	15	4.2%	230	67.3%	7	2.0%	0.61	-0.07	to	0.44
Q52	BI	16	4.5%	191	56.0%	9	2.6%	0.64	-0.29	to	0.33
Q53	SX	86	24.1%	130	48.0%	7	2.6%	0.20	-0.31	to	-0.10
Q54	SX	92	25.8%	164	61.9%	0	0.0%	0.10	-0.30	to	-0.01
Q55	SX	172	48.2%	83	44.9%	60	32.4%	0.39	-0.01	to	0.27
Q56	SX	192	53.8%	64	38.8%	58	35.2%	0.34	-0.10	to	0.25
Q57	SX	199	55.7%	100	63.3%	6	3.8%	0.27	-0.25	to	0.36
Q58	SX	210	58.8%	106	72.1%	8	5.4%	0.28	-0.38	to	0.16
Q59	SX	212	59.4%	56	38.6%	17	11.7%	0.26	-0.28	to	-0.13
Q60	SX	337	94.4%	11	55.0%	2	10.0%	0.15	-0.06	to	0.07

[a] Percentage of the patients with non-missing data

Table B.5 Number of missing responses, floor and ceiling effects, convergent and discriminant validity of the original scale structure of the EORTC-QLQ-BLM30 at T24mo (N=277)

Item	Hypothesized scale	Missings		Floor		Ceiling		Convergent validity	Divergent validity
		N	(%)	N	(%)[a]	N	(%)[a]		
Q31	US	183	66.1%	32	34.0%	7	7.4%	0.75	0.05 to 0.45
Q32	US	185	66.8%	22	23.9%	10	10.9%	0.67	-0.20 to 0.45
Q33	US	184	66.4%	47	50.5%	8	8.6%	0.78	-0.06 to 0.82
Q34	US	186	67.1%	54	59.3%	5	5.5%	0.71	-0.24 to 0.51
Q35	US	184	66.4%	63	67.7%	5	5.4%	0.74	0.14 to 0.65
Q36	US	186	67.1%	55	60.4%	2	2.2%	0.55	0.14 to 0.89
Q37	US	185	66.8%	72	78.3%	2	2.2%	0.41	-0.28 to 0.36
Q38	UP	118	42.6%	118	74.2%	0	0.0%	0.38	-0.03 to 0.18
Q39	UP	120	43.3%	142	90.4%	1	0.6%	0.36	-0.08 to 0.00
Q40	UP	118	42.6%	86	54.1%	1	0.6%	0.34	-0.01 to 0.16
Q41	UP	118	42.6%	131	82.4%	0	0.0%	0.19	0.03 to 0.42
Q42	UP	119	43.0%	116	73.4%	12	7.6%	0.38	0.03 to 0.25
Q43	UP	120	43.3%	108	68.8%	2	1.3%	0.60	-0.07 to 0.31
Q44	CAT	261	94.2%	12	75.0%	0	0.0%		-0.15 to 0.63
Q45	FP	12	4.3%	111	41.9%	8	3.0%	0.79	-0.04 to 0.47
Q46	FP	12	4.3%	134	50.6%	7	2.6%	0.82	0.00 to 0.40
Q47	FP	11	4.0%	159	59.8%	4	1.5%	0.79	0.07 to 0.48
Q48	BAF	10	3.6%	221	82.8%	0	0.0%	0.32	-0.07 to 0.54
Q49	BAF	15	5.4%	136	51.9%	1	0.4%	0.32	0.04 to 0.24
Q50	BI	11	4.0%	167	62.8%	4	1.5%	0.69	0.15 to 0.43
Q51	BI	13	4.7%	170	64.4%	6	2.3%	0.62	0.09 to 0.47
Q52	BI	12	4.3%	145	54.7%	10	3.8%	0.60	-0.10 to 0.32
Q53	SX	75	27.1%	93	46.0%	2	1.0%	0.05	-0.46 to 0.11
Q54	SX	77	27.8%	121	60.5%	0	0.0%	0.04	-0.43 to 0.09
Q55	SX	129	46.6%	61	41.2%	54	36.5%	0.45	-0.08 to 0.21
Q56	SX	148	53.4%	50	38.8%	46	35.7%	0.43	-0.12 to 0.21
Q57	SX	157	56.7%	78	65.0%	7	5.8%	0.12	-0.07 to 0.52
Q58	SX	166	59.9%	83	74.8%	3	2.7%	-0.03	-0.22 to 0.34
Q59	SX	167	60.3%	33	30.0%	11	10.0%	0.17	-0.40 to -0.15
Q60	SX	262	94.6%	4	26.7%	4	26.7%	0.43	-0.14 to 0.56

[a] Percentage of the patients with non-missing data
